# HCTNet: A Hybrid ConvNet-Transformer Network for Retinal Optical Coherence Tomography Image Classification

**DOI:** 10.3390/bios12070542

**Published:** 2022-07-20

**Authors:** Zongqing Ma, Qiaoxue Xie, Pinxue Xie, Fan Fan, Xinxiao Gao, Jiang Zhu

**Affiliations:** 1 Key Laboratory of the Ministry of Education for Optoelectronic Measurement Technology and Instrument, Beijing Information Science and Technology University, Beijing 100192, China; zqma@bistu.edu.cn (Z.M.); xieqiaoxue@bistu.edu.cn (Q.X.); fan_fan@bistu.edu.cn (F.F.); 2 Beijing Laboratory of Biomedical Testing Technology and Instruments, Beijing Information Science and Technology University, Beijing 100192, China; 3 Beijing Anzhen Hospital, Capital Medical University, Beijing 100029, China; 1502010053@mail.ccmu.edu.cn (P.X.); 122020010363@ccmu.edu.cn (X.G.)

**Keywords:** convolutional neural network, vision transformer, optical coherence tomography, image classification

## Abstract

Automatic and accurate optical coherence tomography (OCT) image classification is of great significance to computer-assisted diagnosis of retinal disease. In this study, we propose a hybrid ConvNet-Transformer network (HCTNet) and verify the feasibility of a Transformer-based method for retinal OCT image classification. The HCTNet first utilizes a low-level feature extraction module based on the residual dense block to generate low-level features for facilitating the network training. Then, two parallel branches of the Transformer and the ConvNet are designed to exploit the global and local context of the OCT images. Finally, a feature fusion module based on an adaptive re-weighting mechanism is employed to combine the extracted global and local features for predicting the category of OCT images in the testing datasets. The HCTNet combines the advantage of the convolutional neural network in extracting local features and the advantage of the vision Transformer in establishing long-range dependencies. A verification on two public retinal OCT datasets shows that our HCTNet method achieves an overall accuracy of 91.56% and 86.18%, respectively, outperforming the pure ViT and several ConvNet-based classification methods.

## 1. Introduction

Retina, the only light sensor of the human eye, converts light information into bioelectric signals and sends them to the brain through the optic nerve [1], thereby playing an important role in human vision. Retinal diseases such as age-related macular degeneration (AMD) and diabetic macular edema (DME) are the leading causes of vision loss and permanent blindness worldwide [2]. More than 80% of vision loss can be prevented and cured [3,4] by accurate retinal screening and appropriate treatment in the early stage. Accurate diagnosis of retinal diseases is essential in clinical practice.

Optical coherence tomography (OCT) [5] is a non-invasive imaging technology. It utilizes low-coherence interferometry to obtain cross-sectional images of biological tissue at microscopic resolutions. Based on the light reflected and backscattered from retinal tissue, the structures of different retinal layers can be visualized on the OCT image. Now, OCT, especially spectral domain OCT (SD-OCT), which has high image quality, plays a pivotal role in ophthalmology because it can identify early disease at treatable time points before visual symptoms and irreversible vision loss occurs [6]. However, manual interpretation and identifying retinal diseases based on the huge amount of OCT images are tedious, time-consuming, and prone to yield subjective results.

With the development of biomedical imaging and sensing techniques, high-performance computers, and artificial intelligence algorithms, intelligent disease diagnosis has become possible. Some automatic diagnosis methods have been proposed to identify retinal diseases based on OCT imaging. The traditional automatic diagnosis methods [7,8,9,10,11] employ machine-learning-related models to perform OCT image classification. They utilize well-designed feature descriptors to extract hand-crafted features and then feed the extracted features into the designed classifier to obtain classification results. Different from the traditional methods based on elaborate hand-crafted features, deep neural networks learn to extract different levels of features directly from the raw data. With the characteristics of translation invariance and locality, deep convolutional neural networks (ConvNets) [12,13] are successfully adopted for retinal OCT image classification [14,15,16,17,18]. Fang et al. [19] proposed a lesion-aware ConvNet to classify retinal OCT images. They first used a lesion detection network to output an attention map and then guide the classification network to weight the contributions of local convolutional representations. An iterative fusion ConvNet combining the current and previous layer features was also proposed to perform the classification among the Drusen (the dry form of AMD), DME, choroidal neovascularization (CNV, the wet form of AMD), and normal OCT images [20]. Thomas et al. [21] proposed a multi-scale and multi-path ConvNet with different classifiers to automate AMD diagnosis. In some studies [22,23,24,25,26], the transfer learning strategy was adopted to classify retinal OCT images based on fine-tuning the classic classification models (e.g., GoogleNet [27], VGG16 [28], ResNet [29]).

ConvNets have become a choice in some OCT image classification tasks. Nevertheless, the performance of ConvNets is generally limited to the difficulties of modeling long-range spatial relations in the biomedical image due to the intrinsic locality of convolution operations. More precisely, each convolutional kernel in ConvNets handles only one local pixel subset in the entire image, causing the network to focus on the local context rather than the global context. Inspired by the great success of Transformer [30] in natural language processing applications, the recently proposed vision Transformer (ViT) [31] uses a self-attention-based model to capture long-range dependencies of image pixels and has achieved promising results in image classification. Following this progress, image-based ViT models are widely adopted for various computer vision tasks [32,33,34]. However, the pure Transformer architecture cannot fully utilize the prior biases existing in the images, such as locality and two-dimensional neighborhood structure, and it is difficult for ViT to extract the low-level features, which form important basic structures in an image (such as edges and corners) [35]. Besides, it was observed that ViT models cannot perform well without large-scale datasets [31]. For example, even with pre-training on ImageNet, the classification performance of ViT is still lower than that of ResNet. Adopting ViT for retinal OCT image classification will be very challenging as the number of images available for training in the medical scenario is relatively scarce.

In this paper, we investigate the feasibility of the ViT model for retinal OCT image classification and propose a hybrid ConvNet-Transformer network (HCTNet), which combines the advantages of Transformer in associating long-range dependencies and the advantages of ConvNet in extracting hierarchical abstract local features. Specifically, to alleviate the dependence on large-scale datasets and solve the problem of ViT in low-level feature extraction, a residual dense block (RDB) is designed to construct a low-level feature extraction (LLFE) module first. Then, instead of the straightforward tokenization from the raw input OCT image, a Transformer branch (T-branch) is used to extract patches from the low-level features generated by LLFE and capture global context by leveraging the self-attention mechanism. In addition, as a complement to the T-branch in the HCTNet, a parallel ConvNet branch (C-branch), which also uses RDB as the basic building block, is designed to extract high-level local features. Finally, an adaptive re-weighting-based feature fusion module is attached at the top of the T-branch and the C-branch to achieve the right tradeoff between the global and local context for retinal disease classification. To the best of our knowledge, the proposed HCTNet is the first method of integrating the strength of ConvNet and ViT for automatic retinal OCT image classification, and the experimental results demonstrate its effectiveness and high performance.

## 2. Materials and Methods

### 2.1. OCT Datasets

**Figure 1:**
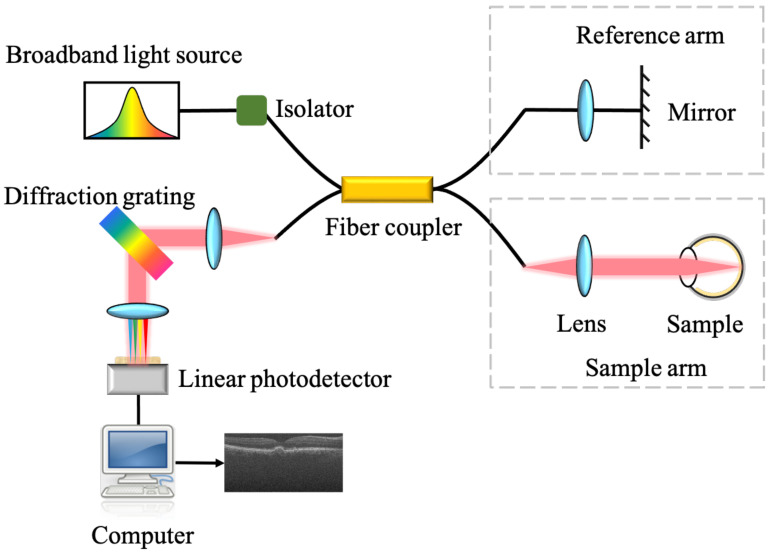
Schematic of the spectral domain OCT system.

The retinal OCT datasets used in this study were acquired by spectral domain OCT (SD-OCT). Figure 1 shows the schematic of a typical spectral domain OCT system [6]. The light from the broad-bandwidth light source is split by a fiber coupler into two beams. One beam is directed onto the tissue sample (e.g., eye) and is back-reflected or backscattered from internal structures at different depths. The other beam is reflected from a reference mirror. The light from the mirror and that from the sample interfere. The interference signal is measured by a spectrometer. The spectrometer uses a diffraction grating to angularly disperse the interference spectrum onto a linear photodetector. The linear photodetector such as a charge coupled device (CCD) or a complementary metal oxide semiconductor (CMOS) detects the spectral interferogram, and an OCT image is generated after further signal processing such as Fourier transform.

Based on the SD-OCT, the OCT2017 dataset [36] and the Srinivasan2014 dataset [37], which were used for evaluation in this study, were obtained from 4686 and 45 patients, respectively. The OCT2017 dataset consists of 84,484 retinal OCT images with the resolution of 512×496 or 768×496, and the images are divided into four classes, i.e., DME, CNV (the wet form of AMD), Drusen (the dry form of AMD), and normal, with the image numbers of 11,598, 37,455, 8866, and 26,565, respectively. The Srinivasan2014 dataset, originally introduced by Duke University, contains volumetric scans acquired from 15 normal patients, 15 AMD patients, and 15 DME patients and includes 723 AMD, 1101 DME, and 1407 normal OCT B-scan images with different resolutions.

### 2.2. Proposed HCTNet Method

**Figure 2:**
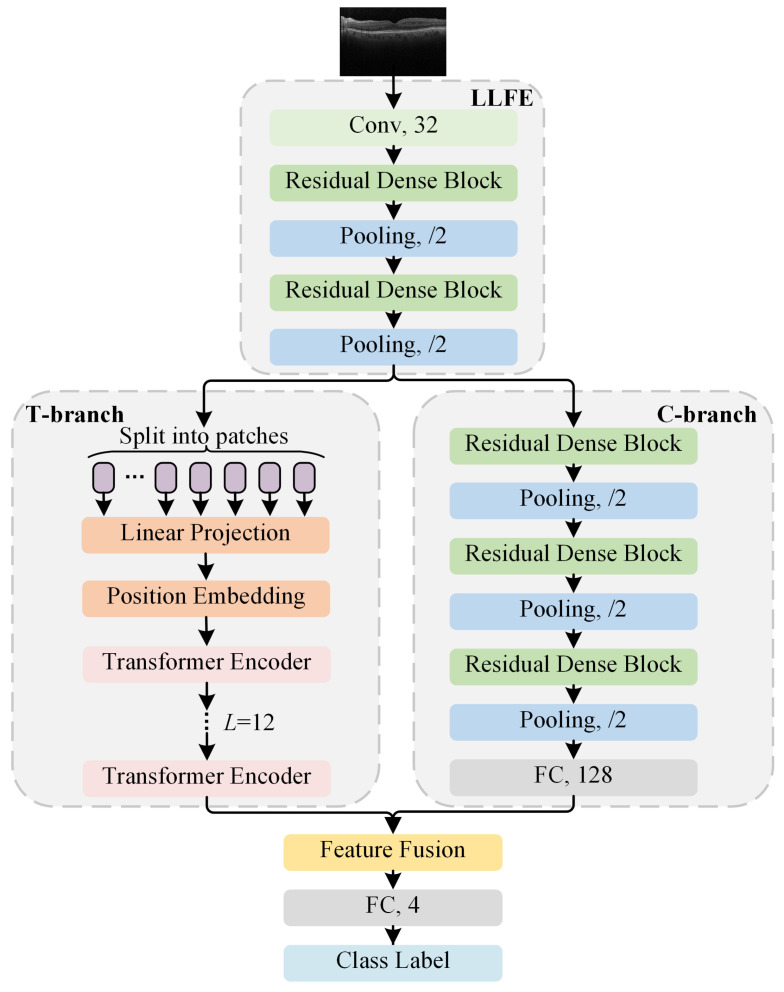
The framework of the proposed HCTNet.

The HCTNet method effectively integrates the advantages of ViT and ConvNet for improving the retinal OCT classification performance. The standard Transformer [30] takes a sequence of token embeddings as the input. To process 2D biomedical images, ViT performs direct tokenization of patches from the raw input image. However, large background areas in the OCT image are meaningless for diagnosis. With straightforward tokenization from the raw OCT image, the irrelevant background area may mislead the model and increase the computational cost. Besides, ViT has much less image-specific inductive bias, which makes it difficult for ViT to extract low-level features (such as edges and corners) in the image and to optimize when trained on a small-scale clinical OCT dataset. To solve these problems, the proposed HCTNet is designed as shown in Figure 2. It takes a retinal OCT image as the input. A well-designed low-level feature extraction (LLFE) module is used to generate low-level features first. Then, two paralleled branches, i.e., the T-branch and the C-branch, exploit the global and local context from the generated low-level features. After that, a feature fusion module is employed to effectively combine the extracted global and local contextual features. Finally, a fully connected layer (FC) is designed for the final retinal disease classification. In the following sections, we introduce the key components of the proposed HCTNet in detail.

#### 2.2.1. Residual-Dense-Block-Based Low-Level Feature Extraction

**Figure 3:**
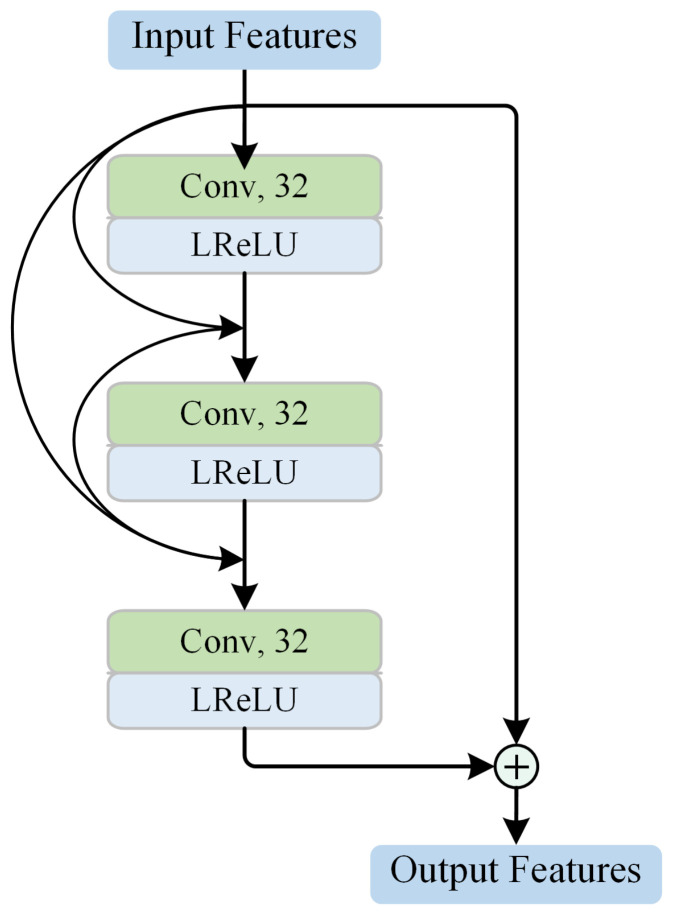
The architecture of the residual dense block.

The LLFE module is designed to generate low-level feature maps. As shown in Figure 2, the LLFE module is composed of one convolutional layer, two residual dense blocks (RDBs), and two pooling layers. The architecture of the RDB is illustrated in Figure 3. The RDB consists of three convolutional blocks, each of which contains a convolutional layer and a leaky rectified linear unit (LReLU). Following the idea of DenseNet [38], each convolutional block connects to every other convolutional block in a feed-forward pattern. To facilitate the network training, the RDB generates the output after employing a residual connection between the input and the last convolutional block. For the pooling layer, max pooling is utilized to make the representation more compact, and the distortion from the irrelevant background areas is ignored. With the RDB and pooling operation, LLFE can take advantage of ConvNet in constructing low-level features and reduce the training difficulty of token embedding by shrinking the patch size.

#### 2.2.2. Transformer Branch for Global Sequence Analysis

Based on the original ViT structure, the key components of the T-branch include tokenization, the encoder module, the multi-head self-attention (MSA) block, and the multi-layer perceptron (MLP) block.

Tokenization: As shown in Figure 2, the T-branch takes the generated feature maps $f\in R^{H\times W\times C}$ of LLFE as input, instead of the straightforward tokenization from the original input OCT image. The T-branch splits the feature maps into *N* ordered 2D patches $f_{p}\in R^{N\times (P^{2}\cdot C)}$, where $N=HW/P^{2}$, (H,W) is the resolution of *f*, *C* is the channel size of *f*, and *P* is the resolution of each patch. Then, the patches are flattened and further processed by linear projection, which employs a trainable matrix $E\in R^{(P^{2}\cdot C)\times D}$ to map the patches into *D*-dimensional embeddings. For the convenience of classification, an extra class token $E_{class}$ is concatenated with the embedded patches, serving as the final representation in the output of the T-branch. Moreover, position embedding $E_{pos}\in R^{(N+1)\times D}$ is appended to the resulting embeddings for reserving the positional information of the patches. The final resulting sequence $z_{0}$ can be denoted as:

(1) $$z_{0}=\left[E_{class};f_{p}^{1}E;f_{p}^{2}E;\cdots ;f_{p}^{N}E\right]+E_{pos}$$

**Figure 4:**
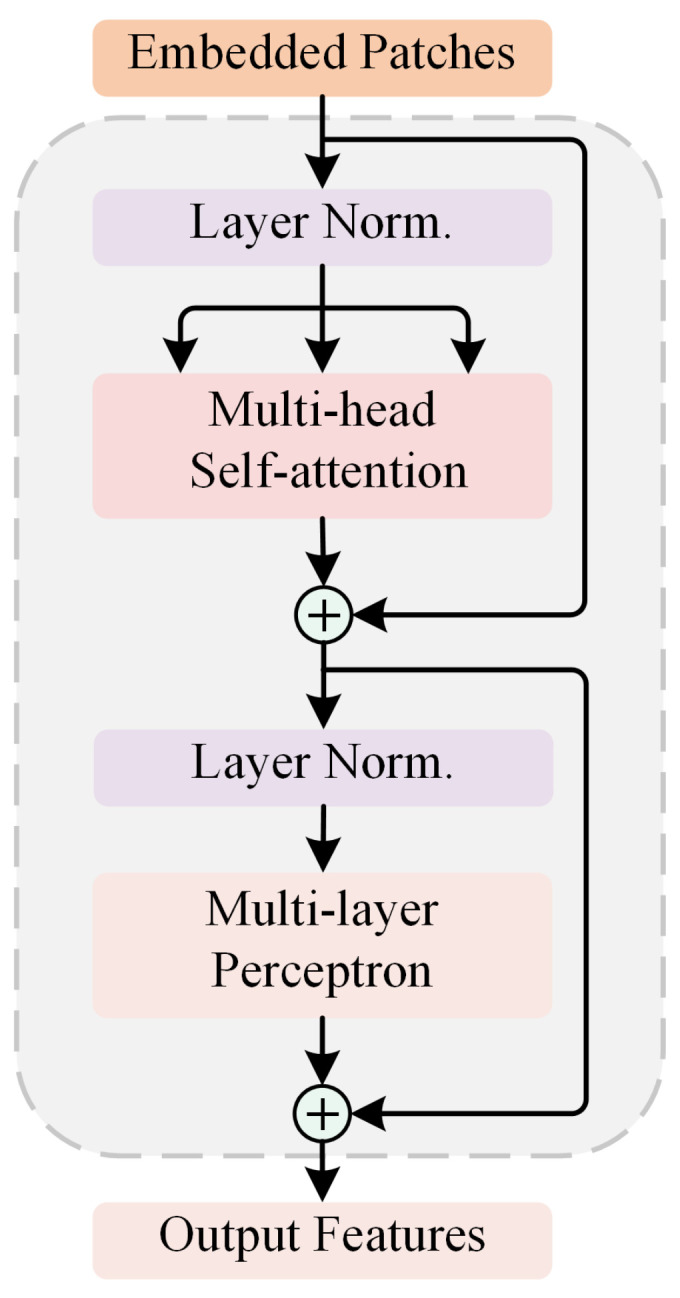
The detailed architecture of the encoder module of the T-branch.

Encoder module: After the above tokenization procedure, the obtained sequence $z_{0}$ is fed into a series of stacked encoder modules. As depicted in Figure 4, each encoder module consists of an MSA block and an MLP block. Layer normalization (LN) and residual connection are applied before and after each block, respectively. Different from ConvNet where feature maps are downsampled at the encoding stage, the output size of each encoder module is the same as its input to ensure the consistency of different encoder modules. The output of the *l*th encoder module $z_{l}$ can be expressed as:

(2) $$\begin{array}{c} z_{l}^{\prime }=\mathrm{MSA}\left(\mathrm{LN}\left(z_{l-1}\right)\right)+z_{l-1}\\ z_{l}=\mathrm{MLP}\left(\mathrm{LN}\left(z_{l}^{\prime }\right)\right)+z_{l}^{\prime } \end{array}$$

where *l* ranges from 1 to *L* and *L* is set to 12. Then, the final output of the T-branch can be obtained by:

(3) $$y=\mathrm{LN}(z_{L}^{0})$$

where $z_{L}^{0}$ indicates the first element of the sequence $z_{L}$ and serves as the image representation.

Multi-head self-attention: MSA, the core component of the Transformer, is an extension of the self-attention block. For the self-attention block, three linear transformations (i.e., fully connected layers) are used to transform the sequence of input tokens $z_{l}\in R^{(N+1)\times D}$ into query matrix $Q\in R^{(N+1)\times D}$, key matrix $K\in R^{(N+1)\times D}$, and value matrix $V\in R^{(N+1)\times D}$, respectively. Then, a weighted sum over all values in the sequence is calculated by:

(4) $$Attention\left(Q,K,V\right)=softmax\left(\frac{QK^{T}}{\sqrt{D}}\right)V$$

where the weight assigned to each value is determined by the normalized scaled dot-product of the query and corresponding key and is then used to adaptively aggregate context information from the values. Therefore, the self-attention block naturally has the global receptive field and the ability to capture long-range dependence. On this basis, MSA further uses the multi-head mechanism to split Q,K, and *V* into several small parts, then performs the attention function on each part in parallel and projects their concatenated outputs to obtain the final output.

Multi-layer perceptron: To enhance the representation ability of tokens, MLP is added after MSA. It performs dimensional expansion/reduction on each token by two linear transformations, with a non-linear transformation in between. The formula of MLP can be written as follows:

(5) $$\mathrm{MLP}\left(x\right)=\sigma \left(xW_{1}+b_{1}\right)W_{2}+b_{2}$$

where $W_{1}\in R^{D\times K}$ and $W_{2}\in R^{K\times D}$ represent the weight values of the two linear transformations, $b_{1}$ and $b_{2}$ are the corresponding biases, and σ(·) denotes the non-linear activation of GELU [39].

#### 2.2.3. ConvNet Branch for High-Level Local Feature Extraction

Although the T-branch is suitable to capture the global and long-range context, scant attention is paid to the locality (neighboring pixels always tend to be correlated) and two-dimensional neighborhood structure of the OCT images. By contrast, with the local receptive field and pooling operation, ConvNets can gradually capture translation-invariant high-level local features and, thus, can help improve classification accuracy. Therefore, a paralleled C-branch is designed in the HCTNet to complement the T-branch, as shown in Figure 2. The C-branch is also built upon the RDB designed in Section 2.2.1. It consists of three alternating RDBs and three max pooling layers and is finalized with a fully connected layer.

#### 2.2.4. Adaptive Re-Weighting-Based Feature Fusion

**Figure 5:**
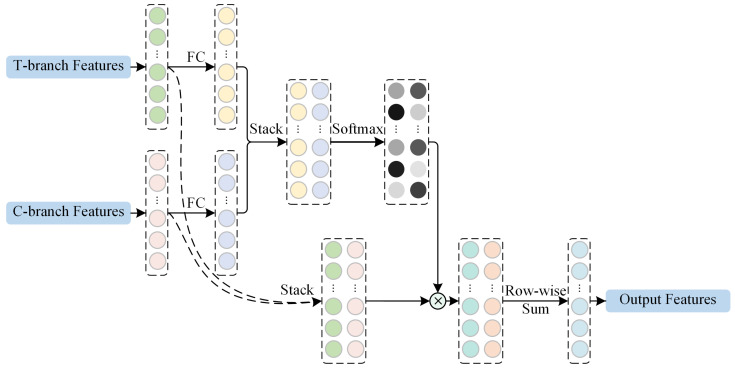
The illustration of the feature fusion module.

As the global features extracted by the T-branch and the high-level local features extracted by the C-branch may contribute differently to the classification of different OCT images, direct fusion will limit the representation ability of the network. Therefore, a feature fusion module is adopted to adaptively emphasize important features for classification. As shown in Figure 5, the feature fusion module first employs two linear transformations to transform the output features of the T-branch and the C-branch. After stacking the data, a softmax function is used to generate a feature aggregating matrix. The feature aggregating matrix is then used as the weight to fuse the global and local features through an elementwise product and a rowwise summation. With the re-weighting mechanism provided by the feature aggregating matrix, the local and global features are adaptively fused according to the input content and can result in better representations for OCT image classification.

### 2.3. Loss Function

The output features of the feature fusion module are processed by a fully connected layer to obtain the final classification result. To train the proposed HCTNet, we adopt the multi-class cross-entropy loss as the loss function for optimization, formally:

(6) $$L_{\mathrm{HCTNet}}=\sum_{x\in \omega }g_{l}\left(x\right)logp_{l}\left(x\right)$$

where ω denotes the set of all training samples, $g_{l}\left(x\right)$ and $p_{l}\left(x\right)$ represent the ground-truth and prediction probability that the input image *x* belongs to class *l*, respectively.

### 2.4. Experimental Protocol

Following the *k*-fold cross-validation strategy, we randomly divided each dataset into *k* equal subsets at the patient level. Considering the size of the OCT2017 dataset, one of the *k* subsets was selected for training and the remaining subsets were used for validation and testing. For the Srinivasan2014 dataset, the subsets were split into 3:1:1 for training, validation, and testing. The experiment on each dataset was performed *k*-times, and the final classification result was achieved by averaging all the testing results.

To generate the training samples, the original images were resized to 224×224 and normalized by subtracting the mean and divided by the standard deviation. Note that no class imbalance optimization strategies were employed in our experiments. In the training procedure, the parameters of the HCTNet were initialized with the Xavier algorithm [40]. The Adam optimizer [41] with a mini-batch size of 32 and weight decay of 0.0001 was used to optimize the network. The learning rate started from 0.0003 and decreased by a factor of 0.1 every 10 epochs with a StepLR scheduler. The early stopping strategy was adopted to avoid the risk of overfitting.

The HCTNet architecture was implemented using PyTorch [42]. We used NVIDIA DALI as the data loader to accelerate the training speed. All experiments were performed on a machine running with an Intel Core i7-9700 K CPU, NVIDIA GeForce RTX 2080 Ti GPU, and 32 G RAM.

### 2.5. Evaluation Metrics

In our experiments, the classification performance of retinal OCT images was evaluated based on the accuracy, sensitivity, and precision. Let $TP_{i}$, $FP_{i}$, $FN_{i}$, and $TN_{i}$ represent true positive, false positive, false negative, and true negative of the *i*th class, respectively, then the metrics are defined as follows:

(7) $$Accuracy_{i}=\frac{TP_{i}+TN_{i}}{TP_{i}+TN_{i}+FP_{i}+FN_{i}}$$

(8) $$Sensitivity_{i}=\frac{TP_{i}}{TP_{i}+FN_{i}}$$

(9) $$Precision_{i}=\frac{TP_{i}}{TP_{i}+FP_{i}}$$

The above metrics are computed for each independent class, i.e., $Accuracy_{i},\;Sensitivity_{i}$, and $Precision_{i}$ denote the accuracy, sensitivity, and precision of the *i*th class.

Furthermore, according to the multi-class confusion matrix, overall accuracy (OA), overall sensitivity (OS), and overall precision (OP) were calculated to quantitatively evaluate the classification performance over all classes. The symbol *N* denotes the number of the testing samples, and *I* represents the number of classes, then the above metrics are formulated as:

(10) $$\mathrm{OA}=\frac{1}{N}\sum_{i=1}^{I}{TP}_{i}$$

(11) $$\mathrm{OS}=\frac{1}{I}\sum_{i=1}^{I}\frac{TP_{i}}{TP_{i}+FN_{i}}$$

(12) $$\mathrm{OP}=\frac{1}{I}\sum_{i=1}^{I}\frac{TP_{i}}{TP_{i}+FP_{i}}$$

The value of all the above metrics lies in the range of [0,1], and a higher value means better classification performance.

## 3. Results and Discussion

### 3.1. Validation of the Proposed HCTNet

To validate the effectiveness of our method, we compared its performance with four widely used classification approaches: transfer learning [36], VGG16 network [28], ResNet [29], and IFCNN [20]. The transfer learning method was reimplemented based on the InceptionV3 [43], which was pre-trained on ImageNet [44] and fine-tuned by freezing all the convolutional layers and retraining the last fully connected layer. The VGG16 network and ResNet are two classical classification networks; we trained them from scratch and modified the final fully connected layer to four or three neurons for retinal OCT image classification. The IFCNN employs an iterative fusion strategy, which utilizes the current and previous convolutional features, to identify different retinal diseases. The proposed HCTNet along with the reference methods were trained with the same configuration described in Section 2.4.

**Figure 6:**
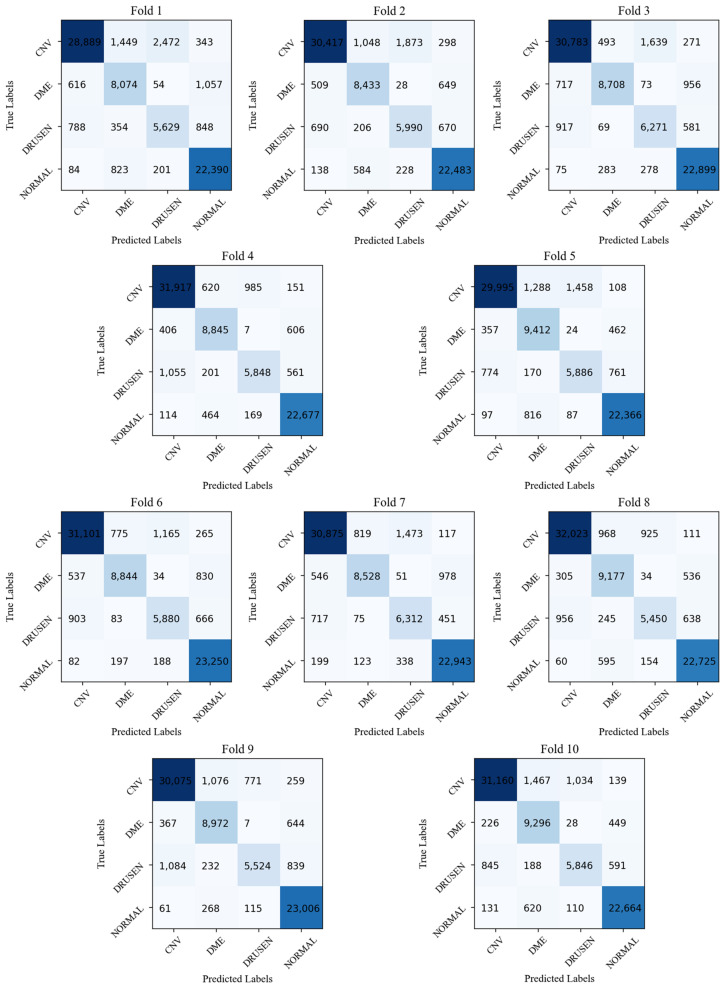
Confusion matrix generated by the HCTNet.

Quantitative evaluation results on the OCT2017 dataset (k=10) are summarized in Table 1, and the confusion matrix of each fold is shown in Figure 6. The proposed HCTNet is superior to the reference methods in terms of the accuracy in all four categories. For the sensitivity metric, the HCTNet yields the best performance in the Drusen, DME, and normal retina classification and ranks second place in the CNV classification. As for the precision, our method achieves the highest value in most categories except for the DME classification, which is 0.18 lower than the highest value achieved by ResNet. Meanwhile, the proposed HCTNet achieves overall classification performance indices with 91.56% accuracy, 88.57% sensitivity, and 88.11% precision, outperforming all the reference methods. Considering the timeliness requirement in clinical applications, the average processing time per image for each method is also shown in Table 1, and the proposed HCTNet ranks in the middle place. Furthermore, we studied the statistical significance of HCTNet’s performance improvement in OA, OS, and OP by the paired *t*-test, and the *p*-values are listed in Table 2, respectively. From Table 2, it can be observed that all the *p*-values are less than 0.05. This means that all improvements in OA, OS, and OP of the HCTNet are statistically significant compared with all the reference methods, demonstrating the effectiveness of the HCTNet for OCT imaging classification.

**Figure 7:**
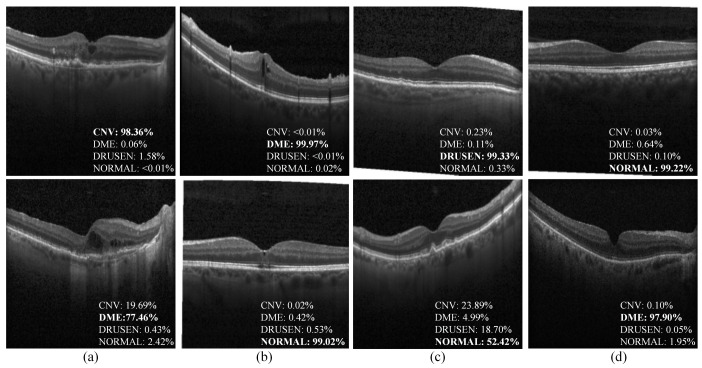
Examples of classification results predicted by the HCTNet on the OCT2017 dataset. The first row shows the good cases, and the second row is the bad cases. (**a**) CNV. (**b**) DME. (**c**) DRUSEN. (**d**) NORMAL.

Figure 7 presents the examples of correct and incorrect classification cases with the predicted probability scores for each class. It can be observed that the HCTNet can correctly predict the categories with a high confidence score. Meanwhile, some misclassification cases occur in the normal and other classes with tiny lesions. Actually, it remains challenging for medical experts to identify the OCT images with tiny lesions. Another misclassification happens between DME and CNV, as they both contain accumulated fluid, which may confuse the proposed HCTNet.

### 3.2. Applicability to Srinivasan2014 Dataset

To evaluate the robustness of our method, experiments were conducted with k=5 on the Srinivasan2014 dataset. The training and testing processes on the Srinivasan2014 dataset were the same as those on the OCT2017 dataset, except that the final output channel of the HCTNet was modified from four to three (as it is a three-category classification problem on the Srinivasan2014 dataset). Table 3 summarizes the quantitative comparisons for different methods on the Srinivasan2014 dataset. For the metrics computed for each independent class, i.e., accuracy, sensitivity, and precision, no one method outperforms all the other methods in all classes, but our method achieves the best performance in four out of nine classification cases and ranks the second or the third place for the non-optimal cases. In terms of the overall evaluation metrics, the superiority of the proposed HCTNet is more remarkable, where the HCTNet performs the best in all metrics and outperforms the second-ranked method with improvements of 1.56%, 3.27%, and 1.06% in OA, OS, and OP, respectively. As for the average processing time, the HCTNet still ranks in the middle place. The results suggest that the HCTNet can be well applied to other relevant OCT datasets, demonstrating its generalization capability.

### 3.3. Robustness to Noise

To validate the robustness of the proposed HCTNet to noise, Gaussian noise was added into the OCT2017 dataset, and the average peak signal-to-noise ratio (PSNR) between the original OCT2017 dataset and its noised version was 26.91. Without retraining the HCTNet on the noised OCT2017 dataset, we directly tested it using the trained model on the original OCT2017 dataset. The quantitative comparison results in terms of overall evaluation metrics are summarized in Table 4. It can be observed that the HCTNet can produce comparable performance on the noisy and original dataset. A further paired *t*-test shows that all the *p*-values are above 0.05. These experimental results demonstrate that the HCTNet is robust to noise to a certain extent.

### 3.4. Ablation Study

**Figure 8:**
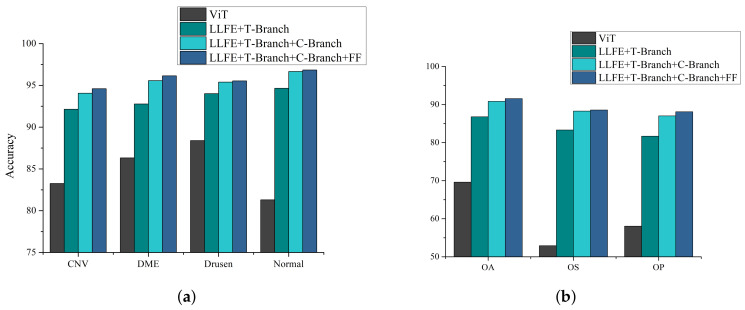
The impact of different components on classification performance. FF denotes the feature fusion module. (**a**) The accuracy metric for each independent class. (**b**) OA, OS, and OP over all classes.

The proposed HCTNet mainly consists of the LLFE module, the T-branch, the C-branch, and the feature fusion module. To evaluate the effectiveness of each module, we gradually added additional modules to the baseline model of the pure Transformer architecture (i.e., ViT [31]) and trained the new models with the same configuration as HCTNet. Then, the accuracy metric for each independent class and the three overall metrics over all classes were calculated to evaluate the impacts of the added module, as shown in Figure 8. The pure Transformer architecture did not achieve satisfactory classification performance, especially in terms of OS and OP. After combining the LLFE, the accuracy of each independent class and the overall classification performance were significantly improved. After further adding the parallel C-branch to the model, better classification performance can be achieved because the two branches can capture complementary information. Finally, the model with the feature fusion module can further improve the model, especially in CNV and DME retina classification, and achieved an OA value of 91.56%. This means that each key component can bring a performance boost. Meanwhile, the results indicate that directly introducing ViT into the small-scale OCT classification task (1/10 used for training) cannot achieve the desired outcome as in the large-scale dataset. By contrast, the well-designed HCTNet can effectively combine the advantages of Transformer and ConvNet and results in superior performance.

## 4. Conclusions

The Transformer architecture has great potential in biomedical disease diagnosis, but its feasibility for OCT image classification remains largely unexplored. In this study, we proposed a hybrid ConvNet-Transformer network (i.e., HCTNet) and verified the feasibility of the Transformer-based architecture for small-scale retinal OCT image classification. By building two parallel branches based on the well-designed low-level feature extraction module, the proposed HCTNet can effectively take advantage of the local feature learning mechanism in ConvNet and the global feature learning mechanism in Transformer. By further incorporating the adaptive re-weighting-based feature fusion module, the feature representation ability of the HCTNet is further enhanced. Our method can be applied to the scenarios where available medical images are limited, and does not require pre-training on the large-scale dataset. The experimental results on the OCT2017 dataset show that our method achieved overall classification performance indices with 91.56% accuracy, 88.57% sensitivity, and 88.11% precision, outperforming the pure ViT and several ConvNet-based classification models. Further verification on the Srinivasan2014 dataset shows that the HCTNet can be easily applied to the other OCT dataset and, thus, has generalization ability and robustness. The HCTNet method can significantly improve the performance of computer-assisted intelligent diagnosis based on retinal OCT images.

## Figures and Tables

**Table 1:** Quantitative comparison results for retinal OCT image classification on the OCT2017 dataset.

Method	Class	Accuracy (%)	Sensitivity (%)	Precision (%)	OA (%)	OS (%)	OP (%)	Time (ms)
Transfer learning [36]	CNV	83.86	92.64	76.52	76.26	57.34	73.47	6.31
	DME	89.53	36.00	74.61				
	Drusen	90.13	18.56	65.22				
	Normal	88.99	92.18	77.55				
VGG16 [28]	CNV	92.92	91.37	92.83	86.68	79.79	81.29	1.08
	DME	94.20	78.79	78.43				
	Drusen	92.34	55.45	65.89				
	Normal	93.90	93.57	88.02				
ResNet [29]	CNV	93.74	90.92	94.92	89.87	86.11	85.82	3.92
	DME	95.88	85.23	84.60				
	Drusen	94.36	72.21	72.74				
	Normal	95.75	96.08	91.01				
IFCNN [20]	CNV	93.45	91.09	94.16	88.67	83.84	84.42	1.46
	DME	95.06	83.68	80.97				
	Drusen	93.95	65.80	72.92				
	Normal	94.8	94.78	89.63				
HCTNet	CNV	94.60	92.23	95.53	91.56	88.57	88.11	3.74
	DME	96.14	87.96	84.42				
	Drusen	95.54	77.36	79.00				
	Normal	96.84	96.73	93.50				

**Table 2:** Statistical analysis (*p*-value) of the proposed HCTNet compared to other networks.

Method	OA	OS	OP
HCTNet & Transfer learning [36]	<$1\times 10^{-4}$	<$1\times 10^{-4}$	<$1\times 10^{-4}$
HCTNet & VGG16 [28]	<$1\times 10^{-4}$	<$1\times 10^{-4}$	0.0002
HCTNet & ResNet [29]	0.0139	0.0038	0.0363
HCTNet & IFCNN [20]	0.0001	<$1\times 10^{-4}$	0.0022

**Table 3:** Quantitative comparison results for retinal OCT image classification on the Srinivasan2014 dataset.

Method	Class	Accuracy (%)	Sensitivity (%)	Precision (%)	OA (%)	OS (%)	OP (%)	Time (ms)
Transfer learning [36]	AMD	90.90	68.37	89.40	79.41	76.25	84.01	6.82
	DME	81.45	76.88	79.10				
	Normal	86.47	83.49	83.54				
VGG16 [28]	AMD	92.76	77.12	86.90	83.69	81.96	85.20	1.30
	DME	84.83	79.76	81.23				
	Normal	89.79	88.99	87.45				
ResNet [29]	AMD	92.35	71.73	90.28	84.55	82.13	86.92	4.02
	DME	87.48	81.41	86.12				
	Normal	89.28	93.26	84.36				
IFCNN [20]	AMD	92.46	71.71	92.49	84.62	81.86	87.47	1.60
	DME	86.54	82.09	83.10				
	Normal	90.24	91.78	86.82				
HCTNet	AMD	95.94	82.60	95.08	86.18	85.40	88.53	3.81
	DME	86.61	80.22	85.29				
	Normal	89.81	93.39	85.22				

**Table 4:** Quantitative comparison results on the noisy and original OCT2017 dataset.

Datasets	OA (%)	OS (%)	OP (%)
Noisy OCT2017	91.52	88.57	88.20
Original OCT2017	91.56	88.57	88.11

## Data Availability

Data underlying the results presented in this paper are not publicly available at this time, but may be obtained from the authors upon reasonable request.
